# Worldwide research trends of post-subarachnoid hemorrhage epilepsy from 1995 to 2024: a bibliometric analysis

**DOI:** 10.3389/fneur.2025.1567068

**Published:** 2025-05-21

**Authors:** Gui Gui, Dajun Yang, Jun Ren, Ruofei Liang

**Affiliations:** ^1^Department of Neurosurgery, Affiliated Hospital of North Sichuan Medical College, Nanchong, China; ^2^School of Clinical Medicine, North Sichuan Medical College, Nanchong, China; ^3^School of Administration, North Sichuan Medical College, Nanchong, China

**Keywords:** bibliometrics, SAH, epilepsy, research hotspots, visualization

## Abstract

**Objective:**

The present study aims to examine the current status, research hotspots, and trends of epilepsy following subarachnoid hemorrhage (SAH) by generating visual maps, and offering research directions and references in the field of post-SAH epilepsy.

**Methods:**

We employed bibliometric methods using VOSviewer, Microsoft Excel, and SRplot to visually analyze data on post-SAH epilepsy from the Web of Science Core Collection (WoSCC). Analysis parameters included the number of papers (NP), countries/regions, institutions, authors, journals, and keywords, assessed through network mapping.

**Results:**

Our analysis included 1,172 publications from 1995 to 2024. The annual NP showed a growing trend, with the United States contributing the highest NP (488) and demonstrating close collaborations with other countries/regions. Harvard University in the United States had the highest institutional output, with 62 papers. The most prolific author was Jan Claassen, with 35 publications, while *Neurocritical Care* was the journal with the highest NP (51). The primary disciplinary category was Clinical Neurology. Keywords such as ‘inflammation,’ ‘prevalence,’ and ‘delayed cerebral ischemia’ (DCI) emerged as recent research hotspots.

**Conclusion:**

Over the past three decades, there has been a significant upward trend in the annual NP on post-SAH epilepsy. The United States has maintained a leading position in this field. Current research primarily focuses on the pathogenesis, with particular attention to ‘inflammation’ and ‘DCI’.

## 1 Introduction

Subarachnoid hemorrhage (SAH) is a life-threatening condition most commonly caused by the rupture of intracranial aneurysms (1). According to the WHO MONICA Stroke Study, the overall 28-day mortality rate for SAH patients is 42% (2). Epilepsy, a common complication following SAH, has an incidence ranging from 4.2 to 26% (3–5). Given its negative impact on long-term survival and cognitive recovery after aneurysmal rupture, post-SAH epilepsy has attracted increasing research attention (6). Herein, we employed a bibliometric strategy to analyze and visualize the current status and research hotspots in post-SAH epilepsy. Notably, bibliometrics is a quantitative method used to analyze the literature within a specific field (7). In recent years, it has been widely applied in the medical field, including studies on delayed cerebral ischemia (DCI), neurogliomas, and cerebral vasospasm (8–10). However, to date, no bibliometric studies specifically focusing on post-SAH epilepsy have been reported. To that end, we analyzed 1,172 articles published in this field over the past 30 years, as indexed in the Web of Science Core Collection (WoSCC), using tools such as VOSviewer, Microsoft Excel, and SRplot for visual analysis.

## 2 Methods

### 2.1 Data source and search strategy

**Figure 1:**
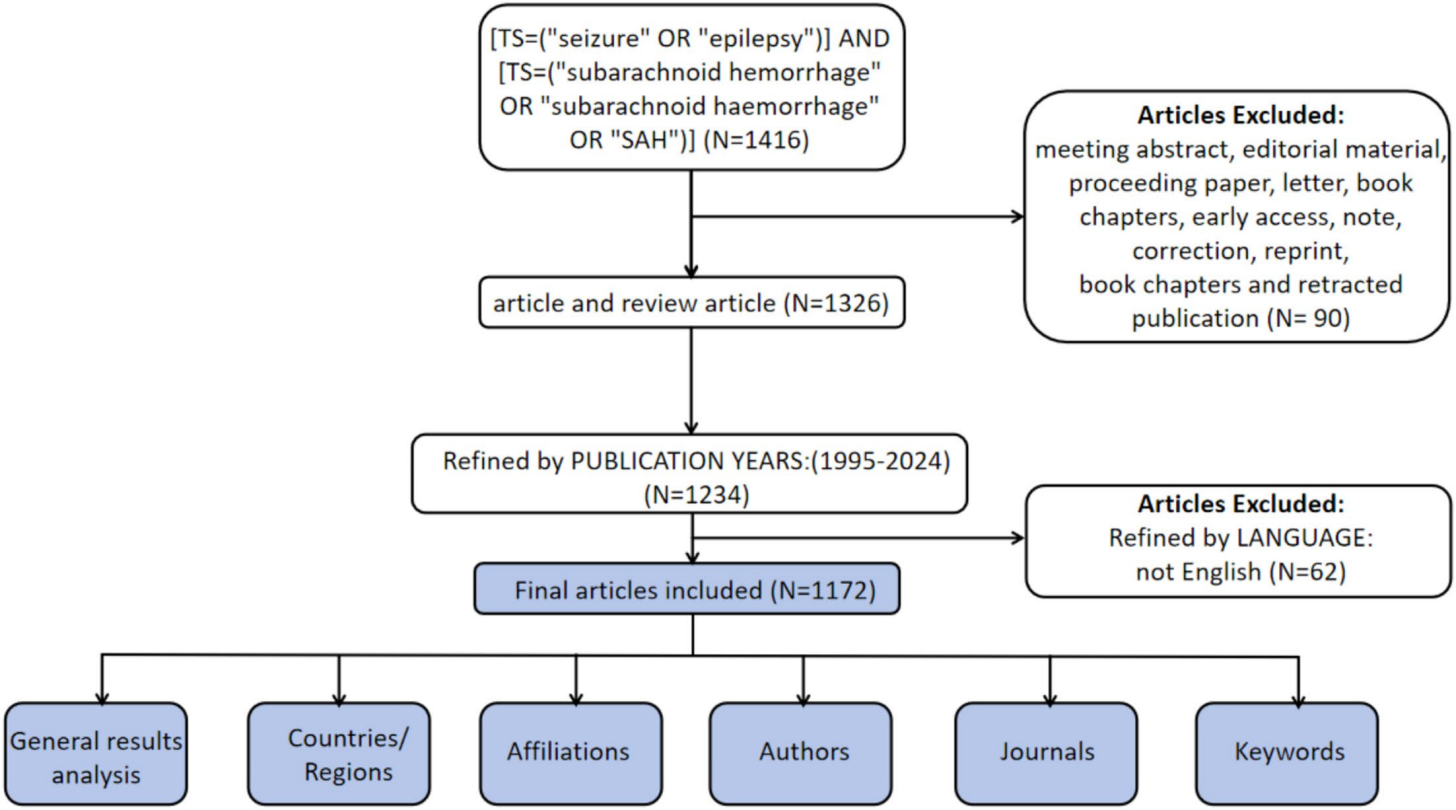
Article inclusion flowchart.

The WoSCC is recognized as a high-quality digital literature database and has been widely used by researchers for bibliometric analyses (11). Therefore, WoSCC was selected as the data source for the present study. To minimize potential bias from ongoing database updates, literature retrieval and data extraction were performed on a single day (January 23, 2025). The search strategy was as follows: [TS = (“seizure” OR “epilepsy”)] AND [TS = (“subarachnoid hemorrhage” OR “subarachnoid haemorrhage” OR “SAH”)]. Inclusion criteria were limited to English publications and document types defined as ‘article’ and ‘review article’ published between January 1, 1995, and December 31, 2024. A total of 1,172 publications met the inclusion criteria (Figure 1).

### 2.2 Data analysis

After exporting the literature data from the WoSCC in TXT format, we used VOSviewer (version 1.6.20, October 31, 2023) for visual analysis. By constructing bibliometric network maps, we visually revealed the interconnections among publications and analyzed research hotspots and trends. Each item, such as keywords, countries/regions, institutions, and authors, was represented by a node in the network. The size of each node was proportional to the number of papers (NP) associated with it, while the thickness of the lines connecting nodes indicated the strength of relationships, such as co-authorship between countries/regions, with thicker lines denoting stronger collaborations. In addition, we conducted a co-authorship analysis of countries/regions, institutions, authors, and references, using total link strength (TLS) to describe the intensity of links between nodes (12). Different clusters were distinguished by different colors (13). Information such as NP, number of citations (NC) excluding self-citations, H-index, average publication year (APY), country/region, affiliation, author, journal, reference, category, and keywords was counted and ranked using Microsoft Excel (.xlsx) and SRplot (http://www.bioinformatics.com.cn/SRplot, accessed 23 January 2025), and visualized using charts (14). Scatter plots were generated using Microsoft Excel, while bar graphs, heatmaps, and donut charts were produced using SRplot.

## 3 Results

### 3.1 General results analysis

**Figure 2:**
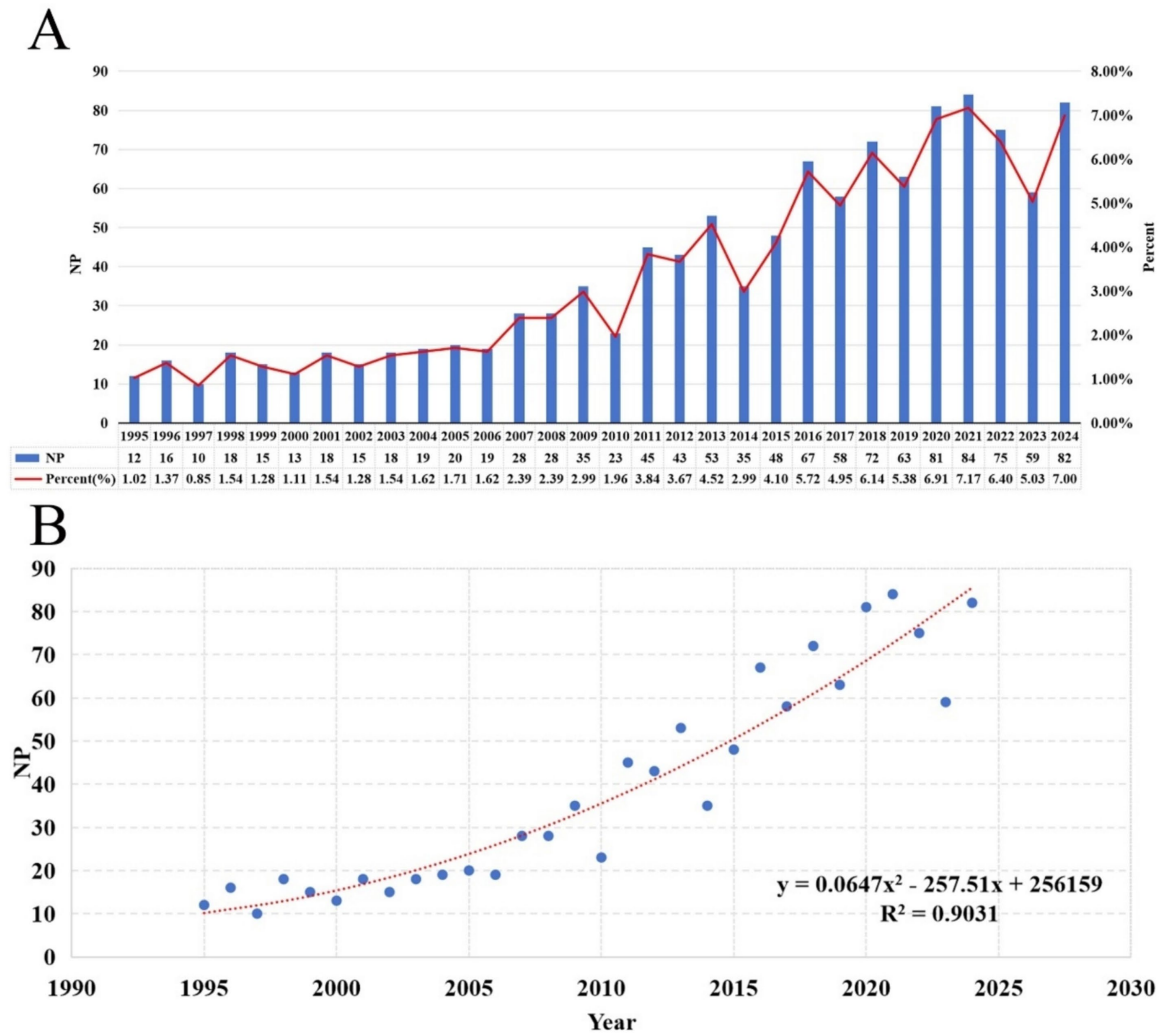
**(A)** Annual NP and the percentage per year over the past 30 years. **(B)** Polynomial fit curves for annual NP trends.

Following our search strategy, we included 1,172 publications, with a total NC of 34,976, an average NC of 32.43, and an H-index of 94. The NP was highest for 2021, standing at 84 (7.17%) (Figure 2A). Over the past 30 years, there has been an upward trend in annual NP, with a significant correlation between annual NP and the year of publication. The polynomial fitting curve yielded a correlation coefficient (*R*^2^) of 0.9031 (Figure 2B).

### 3.2 Analysis of countries/regions

**Figure 3:**
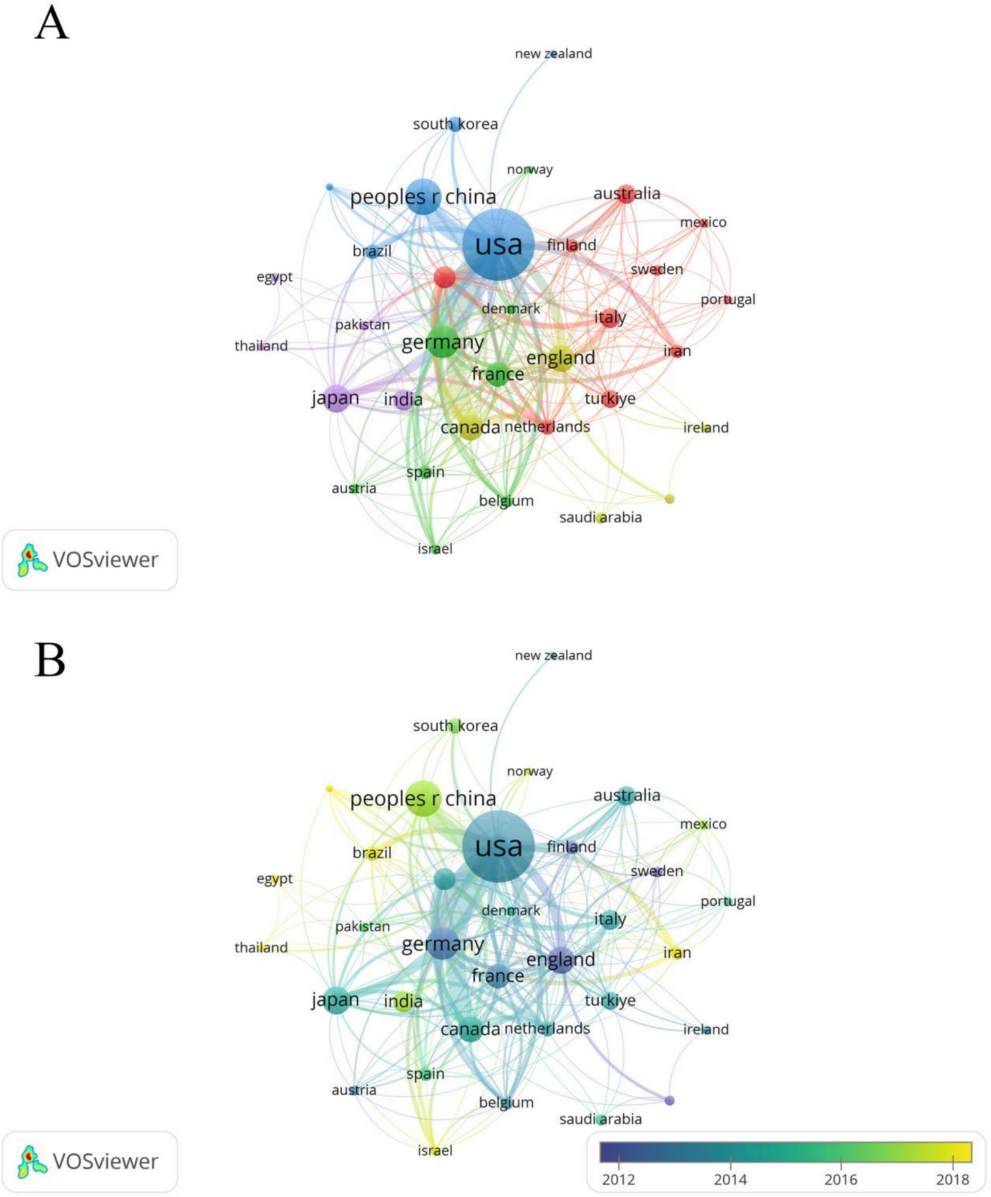
**(A)** Cooperation network between countries/regions. Among the 94 countries/regions, 36 had at least 5 documents. **(B)** Time visualization of countries/regions according to the APY.

**Figure 4:**
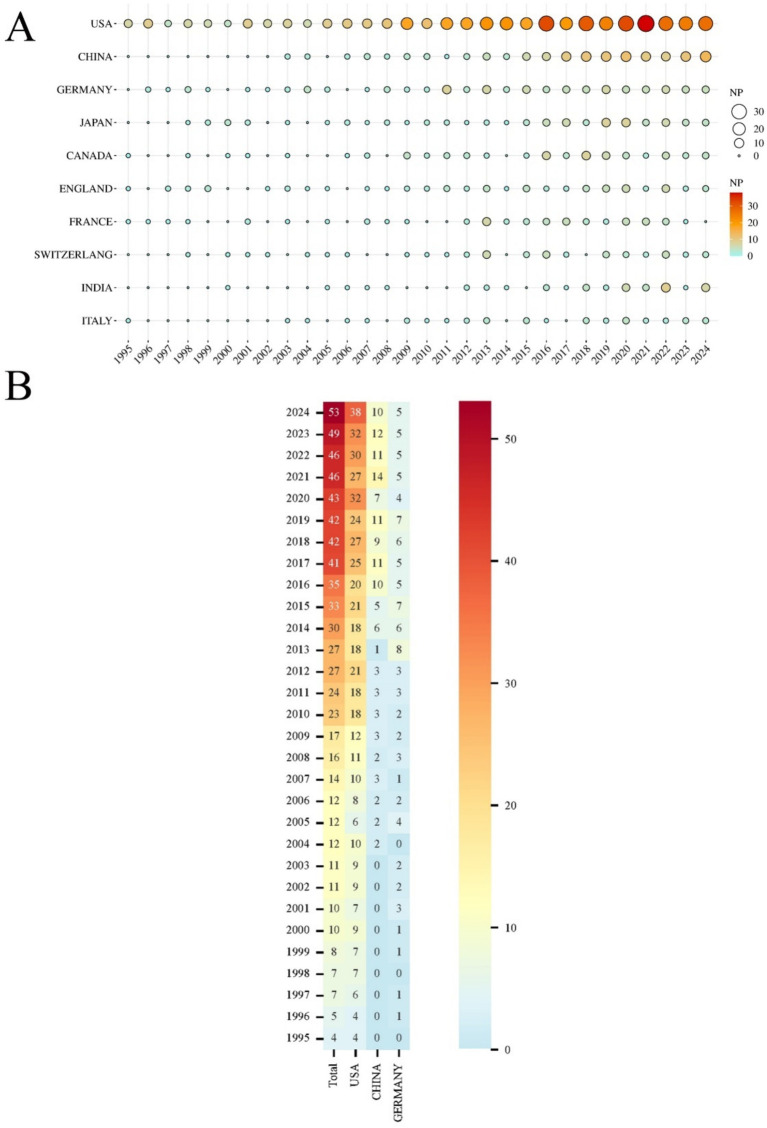
**(A)** Top 10 countries/regions in annual NP over the past 30 years. The size and color of the circles represent the annual NP; the larger the circle and the deeper the color (from yellow to red), the higher the NP and the greater the research focus in that year. **(B)** Heatmap of the top 3 countries/regions (USA, China, and Germany) in annual NP of post-SAH epilepsy over the past 30 years. The color of each box indicates the NP, with darker red representing a higher NP and blue indicating an NP close to zero.

A total of 94 countries/regions have published relevant literature in this field. The United States leads with the highest NP (488) and NC (17,332), followed by China, which ranks second in NP (NP = 130, NC = 2,416), but with significantly lower NC compared to Germany (NP = 99, NC = 4,024) (Table 1). The United States also has the most extensive collaborations with other countries/regions, with a TLS of 222 (Figure 3A). The United Kingdom (APY = 2013.5625), Sweden (APY = 2007.5833), and Finland (APY = 2012.2941) are among the earlier researchers in this field (Figure 3B). China’s earliest publications in this area date back to 2003, with a relatively recent APY of 2017.6462. Since 2017, China has consistently published no fewer than 10 papers annually (Figures 4A,B), marking it as an emerging force in this research area.

**Table 1:** Top 10 countries/regions by publication volume.

Rank	Countries	% of (1172)	NP	NC	H-index
1	USA	41.64%	488	17,332	70
2	CHINA	11.09%	130	2,416	28
3	GERMANY	8.45%	99	4,024	33
4	JAPAN	6.40%	75	1,725	22
5	CANADA	5.63%	66	2,605	25
6	ENGLAND	5.55%	65	2,433	24
7	FRANCE	4.69%	55	3,451	28
8	SWITZERLAND	4.01%	47	1,490	20
9	INDIA	4.01%	47	586	11
10	ITALY	3.16%	37	1,323	19

NP, number of papers; NC, number of citations.

### 3.3 Analysis of affiliations

**Figure 5:**
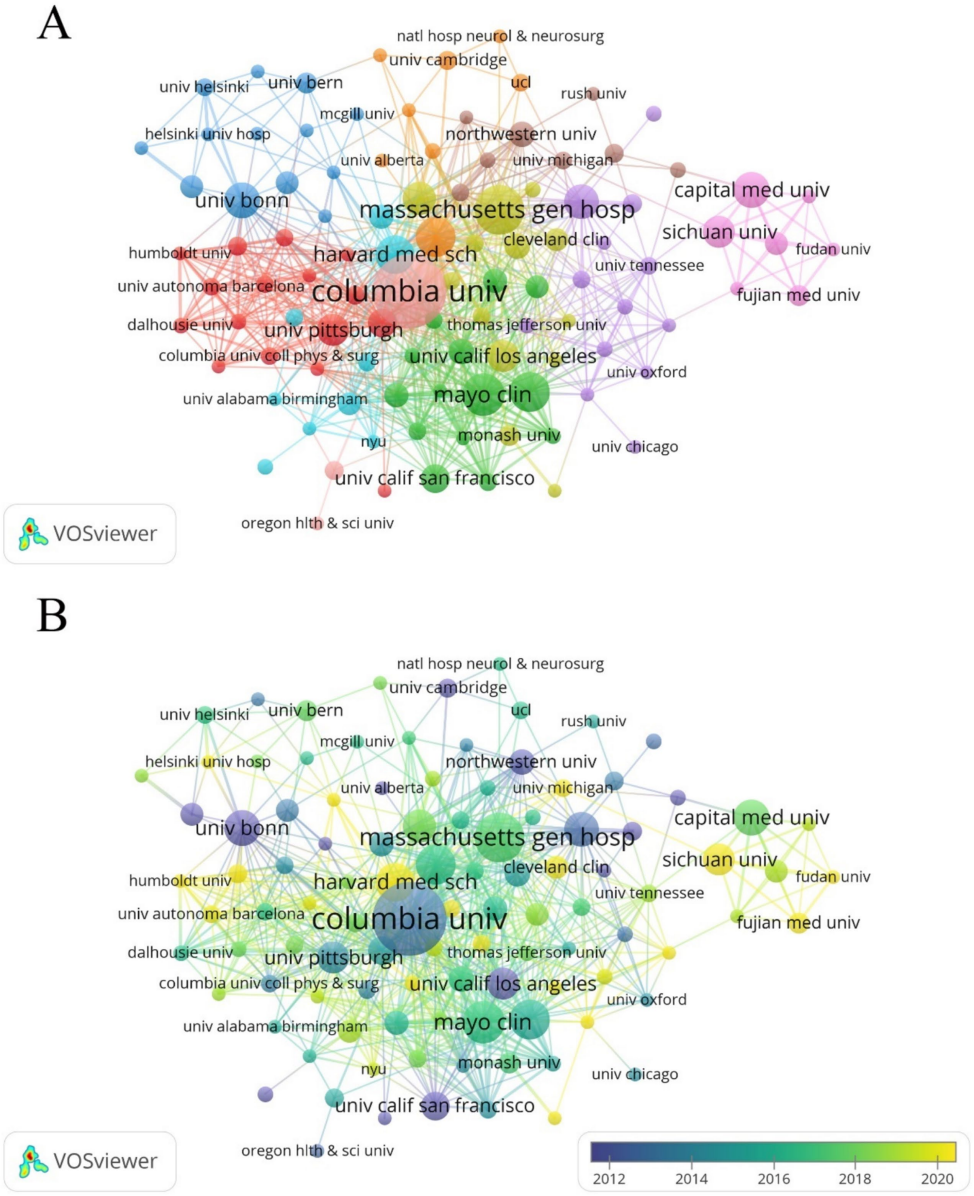
**(A)** Cooperation network between affiliations. Among the 1,764 affiliations, 110 had at least 5 documents, and 109 of these had cooperative relationships. **(B)** Time visualization of affiliations according to the APY.

Harvard University has the highest NP (62), while Columbia University leads in NC (3,944) and H-index (33). Canada’s University of Toronto (NP = 28), France’s Assistance Publique Hopitaux Paris (NP = 24), and Germany’s University of Bonn (NP = 25) are the most productive institutions in their respective countries/regions (Table 2). The cooperation network of institutions was mapped using VOSviewer, with the Mayo Clinic showing the closest connections with other institutions (TLS = 69) (Figure 5A). The University of Ottawa (APY = 2008), University of Münster (APY = 2008.6), and University of Cambridge (APY = 2009.25) were among the early contributors to this research field (Figure 5B).

**Table 2:** Top 10 institutions ranked by publication volume.

Rank	Affiliations	Country	NP	NC	H-index
1	HARVARD UNIVERSITY	USA	62	2,094	26
2	COLUMBIA UNIVERSITY	USA	60	3,944	33
3	UNIVERSITY OF CALIFORNIA SYSTEM	USA	39	2,447	23
4	JOHNS HOPKINS UNIVERSITY	USA	39	1,327	17
5	MASSACHUSETTS GENERAL HOSPITAL	USA	37	1,302	20
6	HARVARD MEDICAL SCHOOL	USA	36	1,121	19
7	MAYO CLINIC	USA	31	784	15
8	UNIVERSITY OF TORONTO	CANADA	28	1,065	15
9	UNIVERSITY OF BONN	GERMANY	25	1,567	19
10	ASSISTANCE PUBLIQUE HOPITAUX PARIS	FRANCE	24	1,984	16
10	UNIVERSITY SYSTEM OF OHIO	USA	24	1,614	17
10	PENNSYLVANIA COMMONWEALTH SYSTEM OF HIGHER EDUCATION	USA	24	1,037	12
10	YALE UNIVERSITY	USA	24	857	14

### 3.4 Analysis of authors

**Figure 6:**
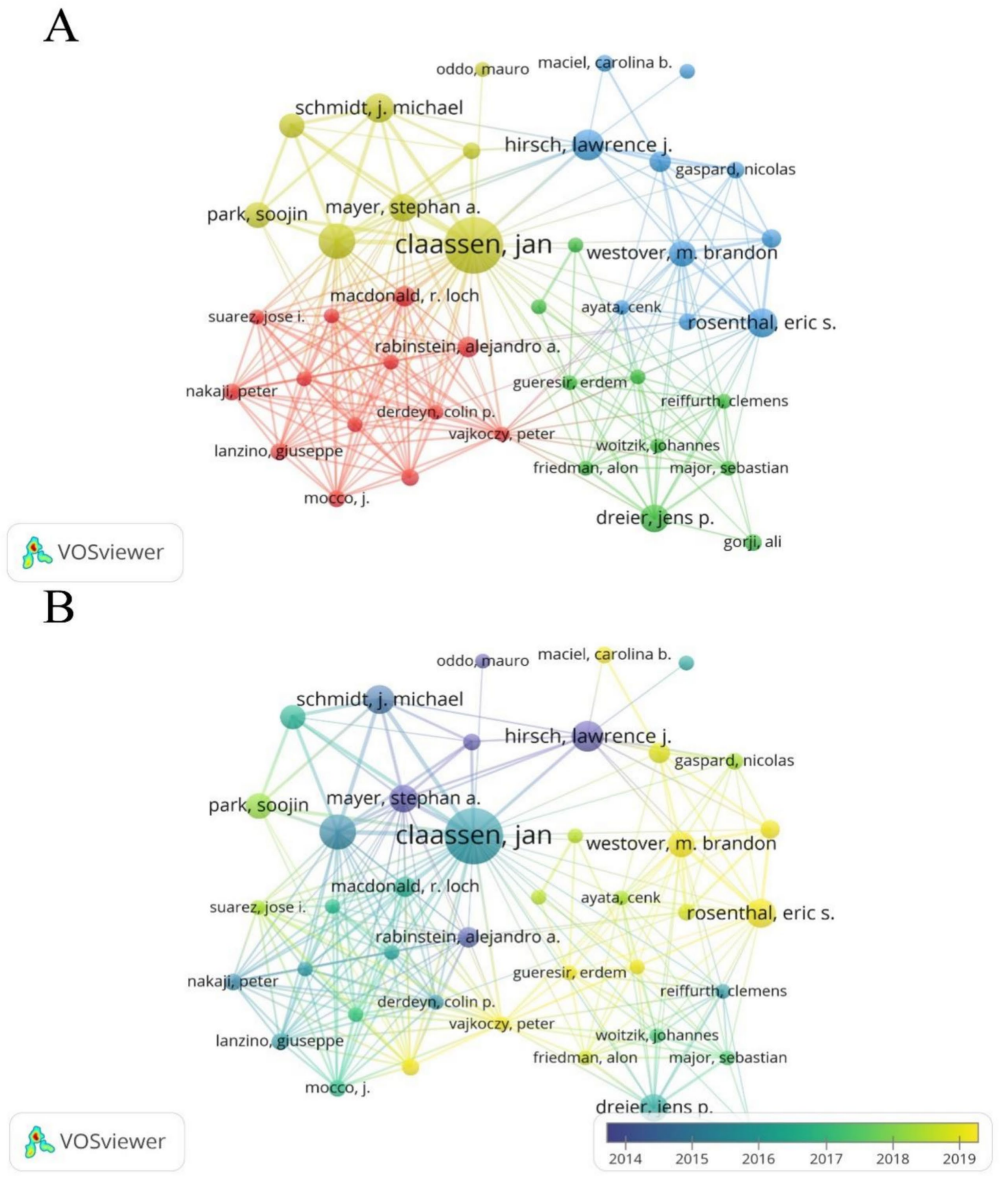
**(A)** Cooperation network between authors. Out of 6,507 authors, 54 had at least 5 documents, and 41 had collaborative relationships. **(B)** Time visualization of authors according to their APY.

Authors from the United States dominate this field and have a significant influence. Among them, Jan Claassen has the highest NP (35), NC (2,797), and H-index (25) (Table 3). Using VOSviewer software, a co-authorship network map was generated, identifying the top three authors in terms of TLS, namely Claassen Jan (TLS = 127), Connolly E. Sander (TLS = 95), and Vajkoczy Peter (TLS = 59). This indicates that these authors are more actively involved in collaborations compared to other researchers (Figures 6A,B).

**Table 3:** Top 10 most productive authors.

Rank	Authors	Country	NP	NC	H-index
1	Claassen, Jan	USA	35	2,797	25
2	Connolly, E. Sander	USA	18	1,366	15
3	Mayer Stephan A.	USA	18	1,375	14
4	Hirsch Lawrence J.	USA	13	1,483	13
5	Dreier, Jens P.	Germany	12	846	10
6	Westover, Michael B.	USA	12	557	10
7	Helmstaedter, Christoph	Germany	11	764	9
8	Rosenthal, Eric Scott	USA	11	547	10
9	Agarwal, Sachin	USA	10	555	8
10	Rabinstein, Alejandro A.	USA	10	343	8
10	Schmidt, J. Michael	USA	10	544	8

### 3.5 Analysis of journals

**Figure 7:**
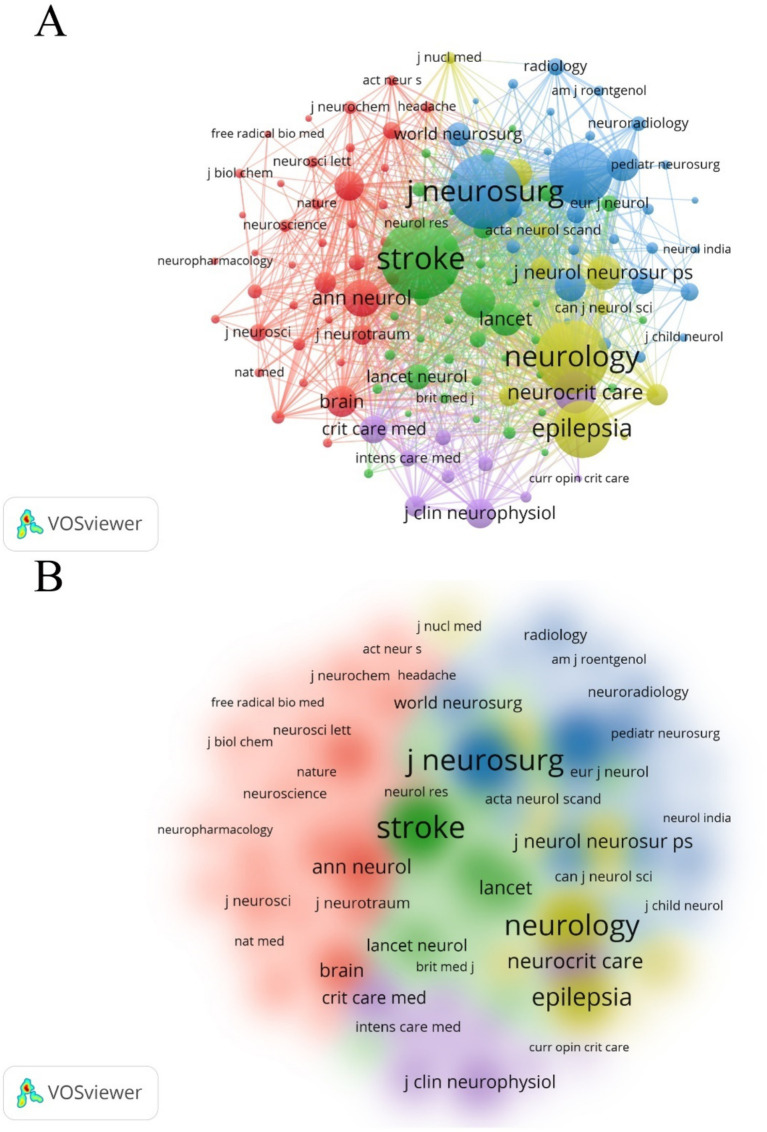
**(A)** Co-citation analysis of journals. The citations for 395 journals totaled 5,222, with 129 journals being co-cited at least 50 times. **(B)** Density map of co-citations of journals.

Table 4 lists the top 10 journals by NP, accounting for 25.26% of the total output (296 out of 1,172). Neurocritical Care ranks highest in NP (51), NC (1,797), and H-index (23). VOSviewer software was also used to generate a co-cited journal network map (Figures 7A,B). The journal *Stroke* received the highest number of co-citations (2,969), followed by the *Journal of Neurosurgery* (2,695), *Neurology* (2,581), *Neurosurgery* (2,027), and *Epilepsia* (1,737).

**Table 4:** Top 10 most-published journals.

Rank	Journals	NP	NC	H-index	*IF (2023)
1	NEUROCRITICAL CARE	51	1,797	23	3.1
2	WORLD NEUROSURGERY	46	528	12	1.9
3	JOURNAL OF NEUROSURGERY	29	1,277	18	3.5
4	NEUROSURGERY	28	1,806	22	3.9
5	NEUROLOGY	28	1,678	20	8.4
6	STROKE	27	3,823	21	7.9
7	JOURNAL OF CLINICAL NEUROSCIENCE	26	325	9	1.9
8	EPILEPSIA	23	1,612	20	6.6
9	CLINICAL NEUROLOGY AND NEUROSURGERY	19	229	9	1.8
10	CUREUS JOURNAL OF MEDICAL SCIENCE	19	28	3	1.0

* IF data sources: journal citation reports 2023 (2024.6.20).

### 3.6 Analysis of co-cited references and categories

**Figure 8:**
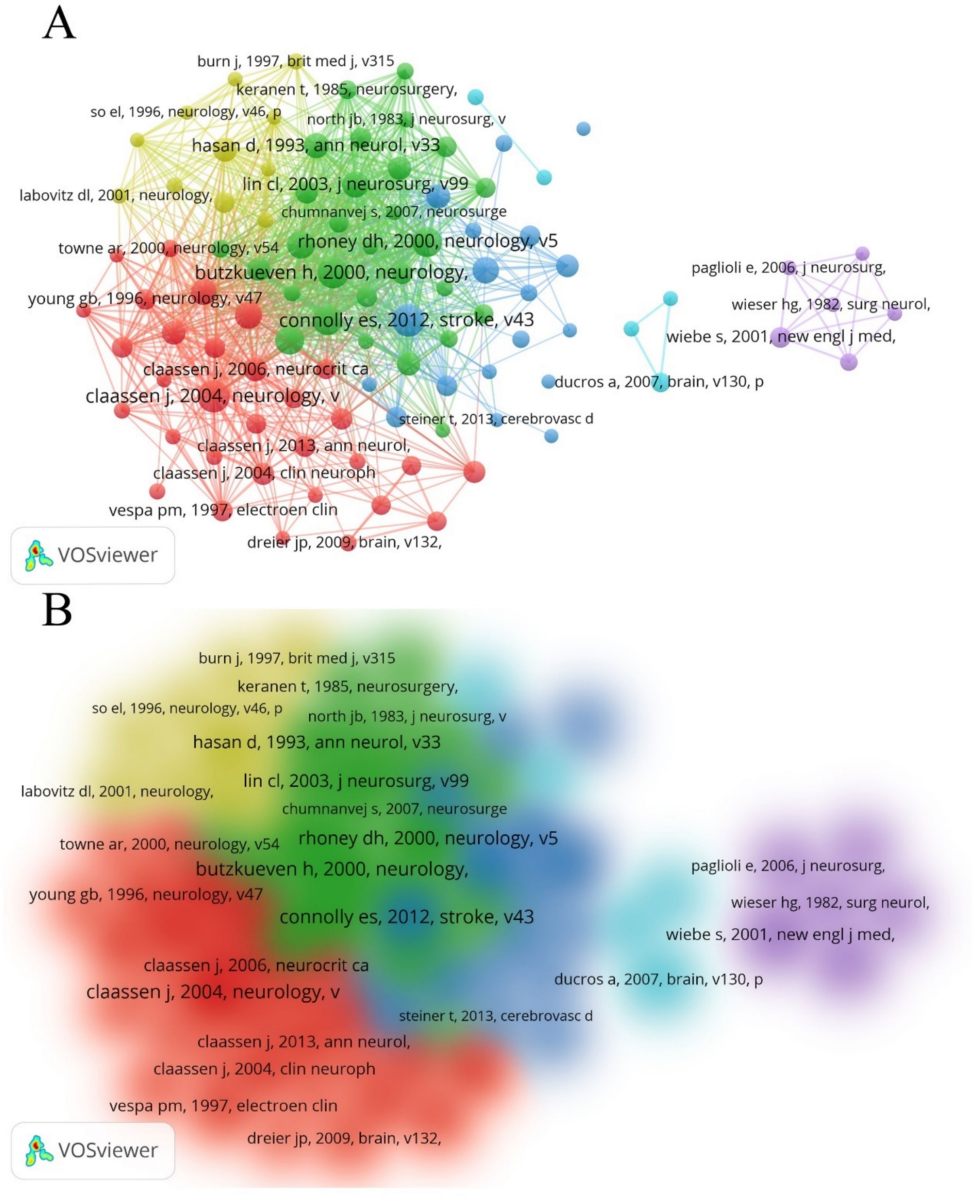
**(A)** Network of co-cited references. Out of all 36,127 references, 100 had been cited at least 20 times. **(B)** Density map of co-cited references.

**Figure 9:**
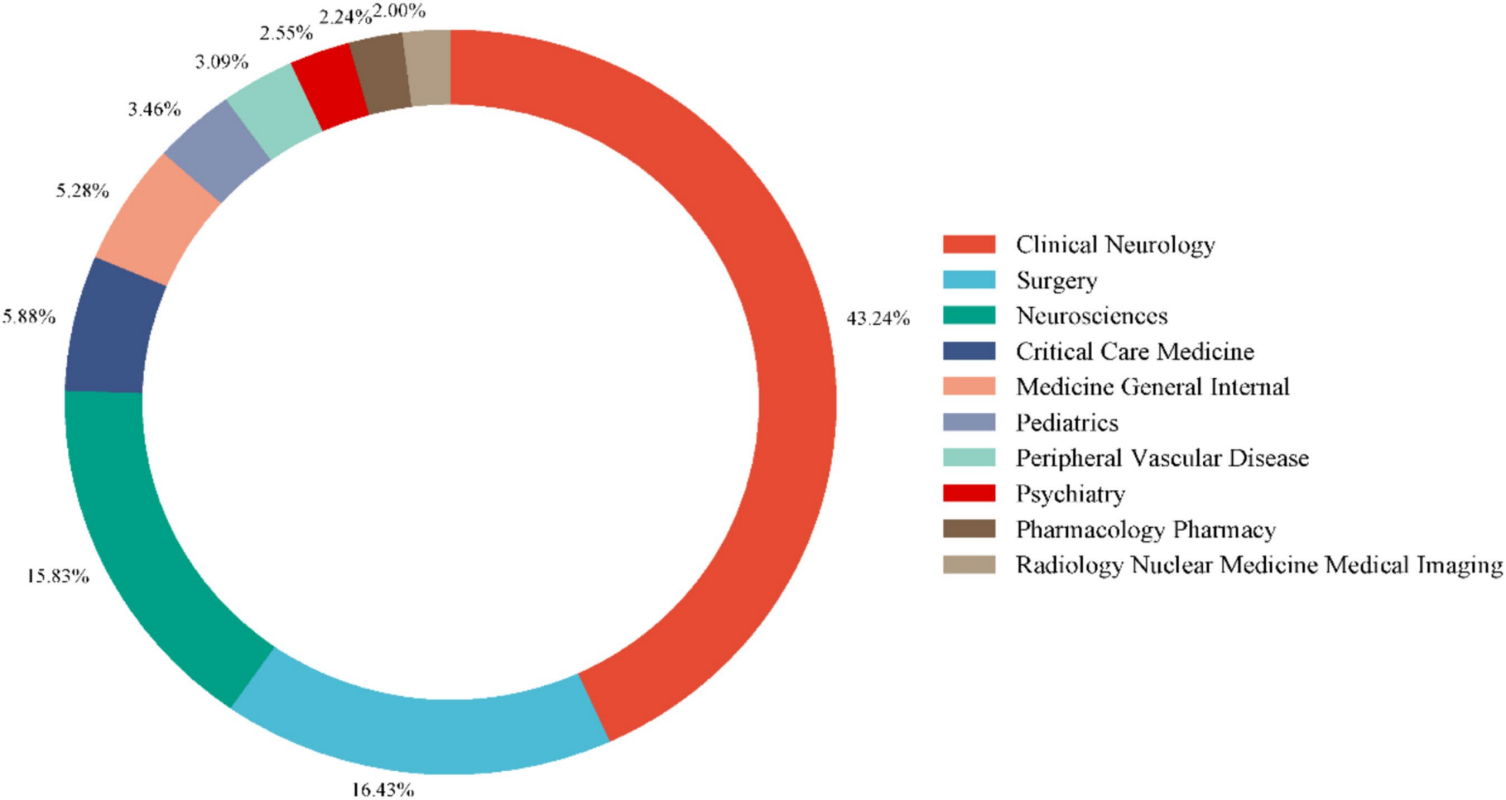
Donut chart of the top 10 research productive categories.

Using VOSviewer, we generated a network map of co-cited references (Figures 8A,B). Among the co-cited references, the publication by Connolly, E. Sander et al. in Stroke (2012) had the highest co-citation frequency, with 83 citations (15). The publication by Butzkueven H. et al. in *Neurology* (2000) had the highest TLS (TLS = 1,030), indicating that this reference has the strongest linkage with other co-cited references (16). The top 10 subjects by NP were led by Clinical Neurology (713/60.84%), followed by Surgery (271/23.12%) and Neurosciences (261/22.27%) (Table 5). Figure 9 displays the proportion of the top 10 research productive categories using a donut chart.

**Table 5:** Top 10 most-published categories.

Categories	Amount	% of (1172)
Clinical Neurology	713	60.84%
Surgery	271	23.12%
Neurosciences	261	22.27%
Critical Care Medicine	97	8.28%
Medicine General Internal	87	7.42%
Pediatrics	57	4.86%
Peripheral Vascular Disease	51	4.35%
Psychiatry	42	3.58%
Pharmacology Pharmacy	37	3.16%
Radiology Nuclear Medicine Medical Imaging	33	2.82%

### 3.7 Analysis of keywords and hotspots in research

**Figure 10:**
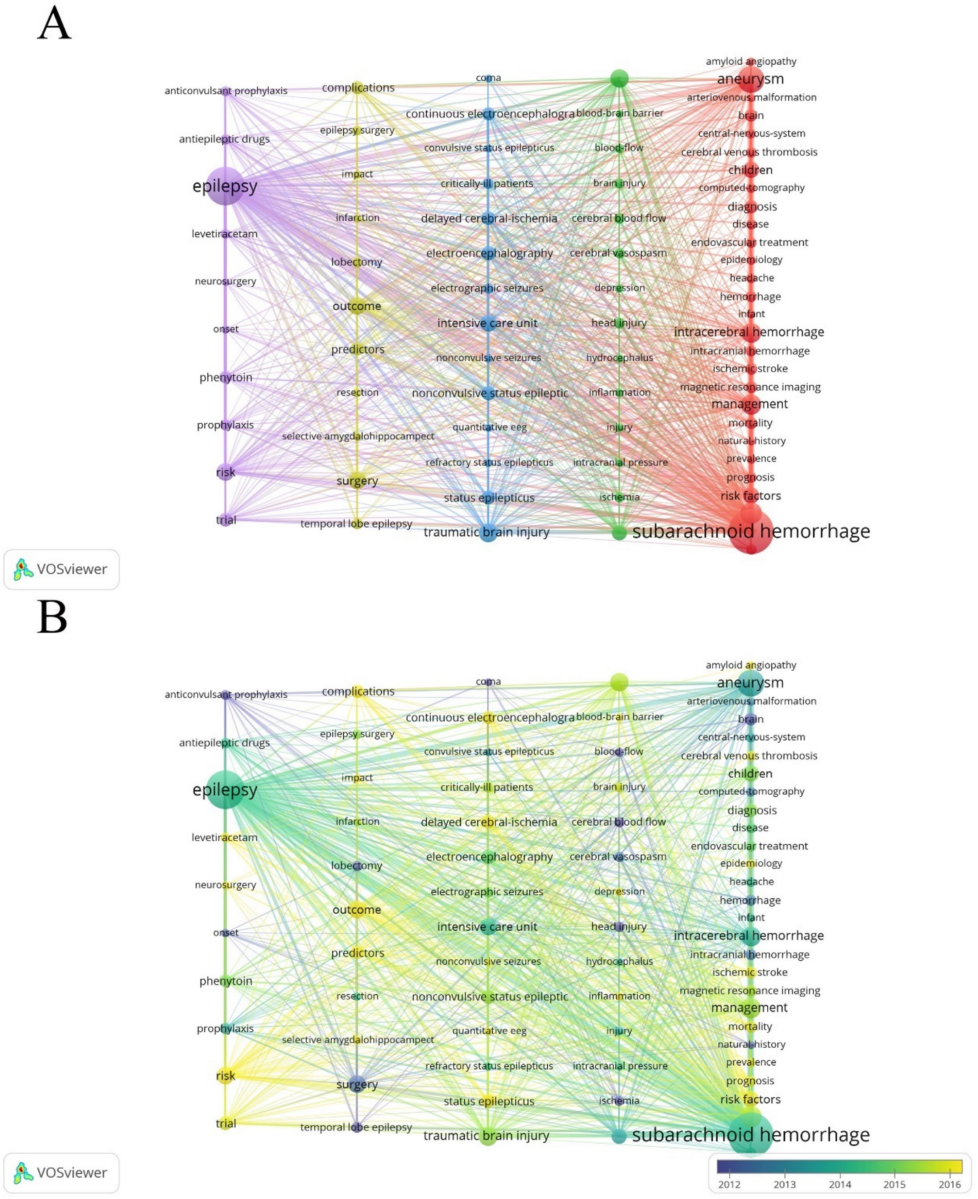
**(A)** Co-occurrence analysis of keywords. Out of a total of 4,553 keywords, 77 appeared at least 20 times after combining similar keywords. **(B)** Time visualization of keywords based on the APY.

Next, we utilized VOSviewer software for frequency statistics and visual analysis of keywords. In addition to the high-frequency keywords ‘subarachnoid hemorrhage’ (572) and ‘epilepsy’ (418), other commonly occurring keywords include ‘aneurysm’ (209), ‘stroke’ (141), and ‘management’ (124) (Table 6). The keywords were divided into five clusters (Figures 10A,B). Cluster 1 (red) focuses on research related to diagnosis, epidemiology, risk factors, and prognosis in post-SAH epilepsy; Cluster 3 (blue) primarily concentrates on types of epileptic seizures and intensive care research; Clusters 2 and 4 (green and yellow) mainly focus on surgical treatments and management; Cluster 5 (purple) emphasizes research on the use of anti-epileptic drugs. Recently emerging keywords include ‘inflammation’ (Cluster 2, APY: 2020.32), ‘prevalence’ (Cluster 1, APY: 2018.069), and ‘delayed cerebral ischemia’ (Cluster 3, APY: 2017.9672) (Figure 10B).

**Table 6:** Top 15 high-occurring keywords.

Rank	Keywords	Occurrences
1	Subarachnoid hemorrhage	572
2	Epilepsy	418
3	Aneurysm	209
4	Stroke	141
5	Management	124
6	Intracerebral hemorrhage	113
7	Traumatic brain injury	112
8	Aneurysmal subarachnoid hemorrhage	104
9	Risk	97
10	Surgery	95
11	Outcome	94
12	Intensive care unit	87
13	Children	84
14	Risk factors	80
15	Vasospasm	73

Herein, we conducted a bibliometric analysis of 1,172 publications on post-SAH epilepsy from 1995 to 2024. Polynomial fitting curves reveal a clear upward trend in the annual NP. The United States leads in NP, NC, and H-index, indicating its dominant authority in this field. China began publishing in this domain in 2003, with a relatively late APY of 2017.6462. Nevertheless, China (NP = 130) ranks second, showing significant and steady research progress with at least 10 publications annually over the past 7 years, emerging as a rising force in this field. Among the top 10 institutions by publication count, the majority are American. Harvard University and Columbia University lead in post-SAH epilepsy research, significantly outpacing other institutions. In terms of disciplinary categories, over half of the publications (713/60.84%) are classified under Clinical Neurology, highlighting the strong relevance and interest in post-SAH epilepsy within this field. Notably, Claassen Jan and his research team from Columbia University (NP = 35, NC = 2,797, H-index = 25) have made significant contributions to the study of post-SAH epilepsy. Their research covers a wide range of topics, from monitoring comatose SAH patients using continuous electroencephalograms (cEEG) to investigating the frequency of epilepsy, its predictive factors, and its impact on patient outcomes, as well as the clinical applications and limitations of cEEG in neuro-monitoring. Before their work, there were no reports in the literature on non-convulsive status epilepticus (NCSE) following SAH. In 2002, Claassen Jan et al. monitored 26 SAH patients using cEEG, with eight of these patients developing NCSE an average of 18 days (range, 5–38 days) after SAH (17). They conducted a prospective analysis of 247 SAH patients, finding an incidence rate of 7% for epilepsy 1 year after SAH, with intracranial hematoma and cerebral infarction identified as predictive factors (18). From 2005 to 2010, Claassen Jan et al. focused on the use of cEEG as a neuro-monitoring tool in clinical settings. They advocated for the use of cEEG monitoring in all neurological ICUs treating refractory status epilepticus (RSE) and suggested its expansion to general ICUs (19–22). In 2014, Claassen Jan et al. confirmed that elevated serum levels of TNF-R1 and hsCRP are associated with non-convulsive seizures (NCSz) during hospitalization (23). Spreading depolarizations (SDs) are waves caused by the sudden, nearly complete disruption of transmembrane ion gradients in neurons and astrocytes (24). Although the precise role of SDs in post-SAH epilepsy is not yet fully understood, early occurrences of SDs have been strongly linked to the development of late epilepsy in patients with aneurysmal SAH (aSAH), suggesting that early SDs may act as biomarkers for the onset of epilepsy (24–26). In 2021, during the COVID-19 pandemic, Claassen Jan et al. used continuous video electroencephalography (cvEEG) to assess the incidence and risk of epilepsy in hospitalized COVID-19 patients, finding two cases of epilepsy resulting from intracranial hemorrhage, including SAH (27). Ongoing research shows that Claassen Jan and his team remain at the forefront of post-SAH epilepsy research, keeping pace with advancements in neurology. Their work has had a significant impact, setting a high standard of excellence in the field.

In recent years, there has been a growing body of basic research on post-SAH epilepsy. Keyword clustering analysis reveals that “inflammation” (cluster 2, APY: 2020.32), “prevalence” (cluster 1, APY: 2018.069), and “delayed cerebral ischemia” (cluster 3, APY: 2017.9672) are the latest research hotspots. The mechanisms of epilepsy are complex and can be influenced by multiple factors, including inflammatory responses, tumors, cerebrovascular diseases, and iatrogenic injuries, all contributing to the high heterogeneity observed in epilepsy (28). As such, exploring the diagnostics and underlying mechanisms of post-SAH epilepsy is particularly important. Researchers have focused on immune-related genes, with inflammation playing a key role in promoting neuronal hyperexcitability, a crucial factor in the development and progression of epilepsy (29).

Neuroinflammation in the central nervous system (CNS) is primarily mediated by microglia, the resident macrophages of the CNS, as the blood–brain barrier (BBB) prevents extensive entry of peripheral immune cells into the brain (30). In the early stages of brain injury, tissue damage may lead to the release of damage-associated molecular patterns (DAMPs), which activate pattern recognition receptors on immune cells, thereby triggering and sustaining the inflammatory response during SAH (31). Under stress, injury, or dysfunction, mitochondrial DNA (mtDNA) can activate inflammation through the cGAS-STING pathway. Oxidized mtDNA (Ox-mtDNA) binds to cytosolic NLRP3, initiating the activation of the inflammasome (32). Shafqat Rasul Chaudhry et al. previously found that levels of specific mtDNA fragments in serum, namely D-Loop and COX-1, were negatively correlated with the risk of epilepsy (31).DCI is a critical factor in the mortality and adverse outcomes of patients with SAH and is closely linked to cerebral vasospasm induced by SAH (33). Moreover, SDs and epileptic seizures may be associated with the onset of DCI following aSAH (34, 35). Both SDs and epileptic seizures are linked to the release of glutamate, which induces neurotoxicity, leading to abnormal neuronal activation and death (36). Therefore, when treating DCI after SAH, patients should be closely monitored for signs of epileptic seizures, and preventive measures should be considered.

By leveraging bibliometric analysis, we provide valuable insights into the research hotspots in post-SAH epilepsy, but our study has several limitations. First, bibliometric analysis primarily relies on quantitative indicators such as the NP, NC, and H-index. Therefore, a comprehensive analysis is needed when interpreting the results, and over-reliance on a single quantitative indicator should be avoided. Second, while bibliometrics helps to identify research trends and hotspots, it does not offer a comprehensive analysis or discussion of the full content of the texts, which may affect the assessment of the actual value and potential of the research. Third, this study used only the Web of Science as the data source and did not include other databases such as PubMed, Scopus, or others. The inclusion criteria and scope of literature databases vary, which may ultimately affect the accuracy and comprehensiveness of the analysis.

## 4 Conclusion

From 1995 to 2024, there has been a clear upward trend in the annual NP on post-SAH epilepsy. The United States has maintained its authoritative position in this field, while China shows significant potential for further research. Currently, the primary focus of discussions on post-SAH epilepsy revolves around the pathogenesis of the disease, particularly in relation to “inflammation” and “DCI”.

## Data Availability

The original contributions presented in the study are included in the article/supplementary material, further inquiries can be directed to the corresponding authors.
